# The Selective 5HT_2A_ Receptor Agonist, 25CN‐NBOH Exerts Excitatory and Inhibitory Cellular Actions on Mouse Medial Prefrontal Cortical Neurons

**DOI:** 10.1002/syn.70014

**Published:** 2025-03-24

**Authors:** Yang Wang, Jesper L. Kristensen, Kristi A. Kohlmeier

**Affiliations:** ^1^ Department of Drug Design and Pharmacology University of Copenhagen Copenhagen Denmark

**Keywords:** action potential, electrophysiological recordings, brain slices, psychedelics, serotonin

## Abstract

Psychedelic compounds have gained renewed interest due to their rapid and long‐lasting therapeutic effects on stress‐related disorders. While the underlying mechanisms of therapeutic actions of psychedelic compounds are still unclear, these drugs are thought to modulate the activity of the serotonergic system, primarily through activating serotonin type 2A receptor (5‐HT_2A_R) and studies have focused on these actions in the medial prefrontal cortex (mPFC). 25CN‐NBOH, a synthetic psychedelic compound with a high binding affinity for 5‐HT_2A_Rs and anti‐anxiety actions, has emerged as a valuable tool for investigating the physiological functions mediated by this receptor. This study aimed to investigate the electrophysiological effects of 25CN‐NBOH on pyramidal mPFC neurons using whole‐cell patch clamp recordings in mouse brain slices. We recorded synaptic events and action potential rates during acute and long‐term exposure to two concentrations of 25CN‐NBOH. Acute application of 10 µM 25CN‐NBOH increased the frequency of spontaneous excitatory postsynaptic currents (sEPSCs) that was reliant on activation of 5‐HT_2A_R, and which was not seen upon chronic exposure. A similar effect of 200 nM 25CN‐NBOH was not noted. Surprisingly, both 10 µM and 200 nM 25CN‐NBOH significantly suppressed the firing rate following acute as well as a longer‐term exposure of 1 h. This suppression was independent of 5‐HT_2A_R activation but was mediated by M‐current channels, as evidenced by the reversal of suppression with the M‐current blocker XE‐991. Our data suggest a complicated dual action of 25CN‐NBOH in enhancing excitatory transmission while also reducing excitability. Our data contribute to knowledge regarding the cellular consequence of 5‐HT_2A_R agonism and contribute to widening our understanding of the potential mechanisms underlying the therapeutic actions of serotonergic psychedelics.

## Graphical abstract

25CN‐NBOH, a synthetic psychedelic compound with high binding affinity for 5‐HT_2A_Rs and anti‐anxiety actions increased sEPSCs and decreased action potential firing of pyramidal mPFC neurons when evaluated in whole‐cell patch clamp recordings in female and male mouse brain slices. Effects on sEPSCs were concentration‐dependent and reliant on 5‐HT_2A_R whereas the effect on action potential firing suppression was independent of 5‐HT_2A_R activation, induced at two different concentrations, and mediated by M‐current channels. These findings suggest a complicated dual action of 25‐CN‐NBOH in enhancing excitatory transmission while also reducing excitability.

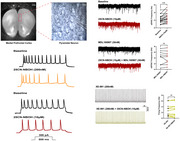

## 1 Introduction

Psychedelics have provided a beacon of hope for mental health treatment as their use in some cases surpasses the clinical effects of traditional therapies, and notably, benefits can manifest more rapidly, which can be especially relevant in the management of treatment‐resistant, major depressive disorder (Nutt et al. 2020; Tullis 2021; Zieba et al. 2022). The cellular and network mechanisms of action of psychedelics are not well understood. Further, conflicting reports, particularly regarding their molecular effects, have emerged making it difficult to know what targets are relevant for therapeutic actions (Moliner et al. 2023). However, it is widely believed that the therapeutic effects are at least partially mediated by agonism at the 5HT_2A_ receptor in neural regions implicated in depressive symptoms (Artigas 2013; Ly et al. 2018; Vargas et al. 2023; Zieba et al. 2022). Further, it is believed that the persistence of therapeutic effects is due to long‐term alterations in function, perhaps due to dendritic plasticity resulting from agonism at the 5HT_2A_ receptor (de Vos et al. 2021; Ly et al. 2018; Martin and Nichols 2018). One of the critical nodes involved in circuits involved in depression is the medial prefrontal cortex (mPFC), and in particular layer 5 pyramidal neurons that express 5‐HT_2A_ receptors appear to play a role. These neurons are modulated by serotonin and play a key role in cognitive function and psychiatric disorders such as depression (Drevets et al. 2008; Koenigs and Grafman 2009; Puig and Gulledge 2011). As fMRI studies of individuals under the influence of psychedelics have suggested that these compounds lead to enhanced activation of mPFC pathways, the prevailing view of cellular actions underlying therapeutic effects of psychedelics includes agonism of 5HT_2A_ receptors in the mPFC (Canal 2018; van Elk and Yaden 2022). Through their agonism of the 5‐HT_2A_ receptors in the mPFC, psychedelics are believed to excite and enhance output from glutamatergic pyramidal neurons to target regions important in mood control and cognitive functioning (Marek 2018; Martín‐Ruiz et al. 2002).

The dramatic therapeutic effects of psychedelics in clinics and data suggesting a role for the 5HT_2A_ receptor have led to a surge in the development of compounds that can selectively activate the 5HT_2A_ receptor on mPFC neurons (Rorsted et al. 2024; G. Zhang and Stackman 2015). Currently, 25CN‐NBOH, a synthetic psychedelic compound belonging to the N‐benzyl phenethylamine class of chemicals, exhibits the highest binding affinity to the 5HT_2A_ receptor of any commercially available tool compound (Fantegrossi et al. 2015; Odland et al. 2021). Behavioral studies of 25‐CN‐NBOH in rodents suggest it possesses cognitive‐enhancing properties, which could include effects at 5HT_2A_ receptors on mPFC neurons (Odland et al. 2021). To date, a peer‐reviewed published study of the cellular effect of 25CN‐NBOH has not appeared. Therefore, we evaluated the cellular effects of 25CN‐NBOH on synaptic and firing activity when acutely applied. In addition, in order to determine whether the effects differed with longer exposures, we evaluated whether effects were seen on synaptic and firing activity following a 1‐h incubation in 25CN‐NBOH.

## 2 Material and Methods

### 2.1 Animals

All experimental procedures were performed in strict accordance with the Danish legislation regulating animal experiments; Law and Order on Animal Experiments; Act No. 474 of 15/05/2014 and Order No. 12 of 07/01/2016. Naval Medical Research Institute (NMRI) mice, an outbred mouse strain bred in‐house from progenitors ordered from Charles River (Denmark) were used. Mice of both sexes were used at 14–30 postnatal days of age and were housed under controlled conditions ensuring maintenance of a fixed temperature (22 ± 1.5°C) and humidity level (55%–65%). Food and water were available ad libitum in a 12 h light/dark cycle (lights on at 7:00 h). All animals were handled using the tunnel method, a well‐established technique known to minimize stress and promote animal welfare during experimental procedures (Gouveia and Hurst 2013).

### 2.2 Drugs

25CN‐NBOH (4‐(2‐((2‐hydroxybenzyl)amino)ethyl)‐2,5‐dimethoxybenzonitrile) was synthesized at the Department of Drug Design and Pharmacology, University of Copenhagen (Hansen et al. 2014). MDL‐100907; XE‐991; AP‐5 (D‐(‐)‐2‐Amino‐5‐phosphonopentanoic acid) and DNQX (6,7‐dinitroquinoxaline‐2,3‐dione) were purchased from Tocris Bioscience. All drugs were bath applied, and electrophysiological recordings were conducted 5 min after the drug reached the bath. To evaluate the persistence of effects, we also conducted an analysis 30 min after drug application and during the period of washout.

### 2.3 Slice Preparation

NMRI mice were anesthetized with isoflurane (Baxter A/S, Denmark). To confirm deep anesthesia, reflexes were tested by gently squeezing the paw with tweezers. Following confirmation, decapitation was performed using surgical scissors, with all procedures conducted on a downdraft table. Immediately after decapitation, the brain was transferred into a slush of ice‐cold artificial cerebrospinal fluid (ACSF) containing (in mM):

124 NaCl, 5 KCl, 1.2 Na_2_HPO_4_•2H_2_O, 2.7 CaCl_2_•2H_2_O, 1.2 MgSO_4_ (anhydrous), 10 dextrose, 26 NaHCO_3_ at pH 7.4 and an osmolality of 298–302 mOsm/kg and was saturated with carbogen (95% O_2_/5% CO_2_). Coronal brain slices with a thickness of 250 µm were obtained using a Leica vibratome (Leica VT1200S, Leica Biosystems, Germany) calibrated before cutting to minimize vertical deflections. The slices were then incubated in a beaker containing oxygenated ACSF at 37°C for 15 min, followed by incubation at room temperature for an additional hour prior to conducting the electrophysiological recordings.

### 2.4 Electrophysiological Recordings

Patch‐clamp recordings were performed using borosilicate patch pipette electrodes with a resistance of 7–11 MΩ when filled with an intracellular solution consisting of (in mM): 144 K‐gluconate, 2 KCl, 10 HEPES, 0.2 EGTA, 5 Mg‐ATP, and 0.3 Na‐GTP, osmolarity range between 295 and 300 mOsM. To record spontaneous inhibitory postsynaptic currents (sIPSCs), a high chloride recording solution was used to optimize the detection of inhibitory events by increasing the drive for, and reversing the polarity of, chloride‐mediated currents. This patch solution contained (in mM): 144 KCl, 0.2 EGTA, 10 HEPES, 0.2 EGTA, 3 MgCl2, 4 Na2ATP, and 0.3 Na‐GTP with an osmolarity between 295 and 300 mOsM. The pipettes were prepared using a horizontal puller (P‐97, Sutter Instruments, USA). Brain slices containing the mPFC were placed in a recording chamber and continuously perfused with carbonated ACSF at 1 mL/min. Individual neurons within the mPFC were visualized using a 60× water immersion objective, which was coupled to an upright microscope (BX50WI, Olympus; Japan) with an infrared Dodt gradient contrast system (IR‐DGC; Luigs & Neumann, Germany) and a CCD camera (CCD‐300ETRC; DAGE‐MTI, Michigan City, IN). Pyramidal neurons were identified based on their morphology and firing patterns (van Aerde and Feldmeyer 2015). A MultiClamp 700B amplifier (Molecular Devices Corporation, USA) was controlled using MultiClamp 700B Commander Software. Recordings were performed in voltage‐clamp mode to establish high‐resistance seals (> 1 GΩ) between the patch pipette and the cell membrane with a holding voltage of −60 mV. Data were collected after membrane breakthrough and a stabilization period of 5 min. Subsequent recordings were conducted using AxoScope 11.2 and digitized at 10 kHz with Axon Digidata 1550B digitizer (Molecular Devices Corporation, USA).

Synaptic events were recorded in voltage‐clamp mode. To record sIPSCs, 50 µM AP‐5 and 15 µM DNQX were included to block glutamate transmission and the patch solution contained high chloride to view sIPSCs as downward deflections. To determine current–voltage (I–V) relationships, a voltage step protocol consisting of incrementing voltage steps ranging from −100 to −30 mV in 10 mV increments was performed, generating an input‐output curve. To monitor action potentials, two protocols in current‐clamp mode were used: the first involved injecting successively increasing currents from 0 to 300 pA in 50 pA increments to trigger neuronal firing. As mPFC cells did not spontaneously fire at rest, the second protocol monitored firing by injecting sufficient current to hold the cell at an identical voltage before and after exposure to 25CN‐NBOH that elicited a barrage of action potentials. Cells were held at the positive potential for 30 s, and after a 10‐s period to allow stabilization of the firing rate, we analyzed the average interval between action potentials in the remaining 20 s. To determine rheobase, in current clamp mode, current injections ranging from 0 to 100 pA in 10 pA increments were applied to identify the voltage of the action potential threshold. For long‐term exposure studies, mPFC slices were incubated with 10 µM 25CN‐NBOH for 1 h before recording.

### 2.5 Statistical Analysis

All data sets derive from recordings taken from at least 4 different mice. In the text, *n* represents the number of neurons, and N_F_ and N_M_ represent the numbers of female and male mice used, respectively. All statistical analyses were performed in GraphPad Prism 10 (GraphPad, Software, USA). The frequency and amplitude of spontaneous excitatory postsynaptic currents (sEPSCs) and spontaneous inhibitory postsynaptic currents (sIPSCs) were analyzed with MINI Analysis software. The firing rate, current–voltage (I–V) curves, and rheobase were evaluated using Clampfit 12.0. All data are presented as means ± standard error of the mean (SEM). Prior to statistical testing, normality was assessed to determine the appropriate analytical approach. For normally distributed data, parametric tests including paired *t*‐tests, unpaired *t*‐tests, and two‐way repeated measures ANOVA were employed. For data that did not meet the normality assumption, nonparametric tests were employed, including the Wilcoxon matched‐pairs signed‐rank test and the Mann–Whitney test. Where applicable, Tukey's test was used for pair‐wise post hoc testing across columns. A *p* value ≤ 0.05 was considered statistically significant, and explicit *p* values are reported in figure legends. A graphical abstract is provided that summarizes the main findings.

## 3 Results

### 3.1 25CN‐NBOH Increases sEPSC Frequency via 5‐HT_2A_ Receptor Activation in Pyramidal Neurons Within the mPFC

**FIGURE 1:**
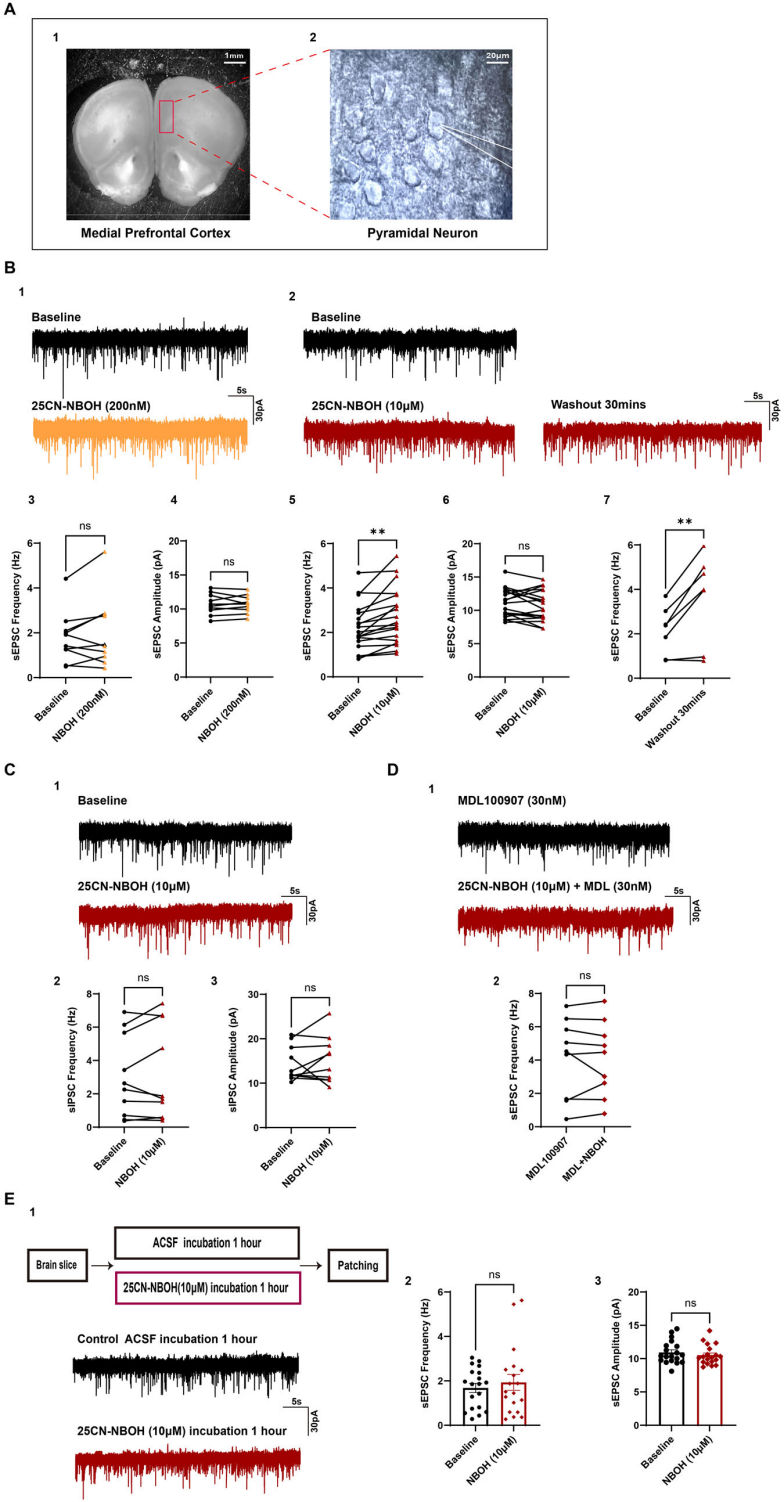
25CN‐NBOH increases the frequency of spontaneous excitatory postsynaptic currents (sEPSCs) through a 5HT_2A_ receptor‐mediated effect when applied acutely; however, incubation in 25CN‐NBOH does not result in a significant frequency change in pyramidal neurons within the mPFC. (A1) Magnified view of a coronal brain slice containing the mPFC under a 10× objective, showing the selected region for recording. (A2) Higher magnification (60× objective) of the recording area at which visualization of pyramidal neurons in the mPFC is apparent. The recording pipette used to conduct electrophysiological recordings from pyramidal mPFC neurons can be seen on the right. (B1) Representative recordings of sEPSCs from a mPFC neuron before and after exposure to bath applied 200 nM 25CN‐NBOH, indicating no significant change in sEPSCs frequency and amplitude. (B2) Representative recording of sEPSCs before and after exposure to 10 µM 25CN‐NBOH in another mPFC neuron, followed by the synaptic activity recorded after 30 min of drug washout. The results indicate an increase in sEPSC frequency, with the effect persisting even after washout. (B3–B4) When evaluating data from the population of recorded cells, there was no significant effect on the frequency (paired *t*‐test, *p* = 0.2655, *n* = 10) or amplitude (paired *t*‐test, *p* = 0.9749, *n* = 10) of sEPSCs in mPFC neurons when exposed to 200 nM 25CN‐NBOH. (B5–B6) However, a significant increase in the frequency of sEPSCs was observed with 10 µM 25CN‐NBOH compared to baseline (paired *t*‐test, *p* = 0.0014, *n* = 19), while no significant change in amplitude was elicited (paired *t*‐test, *p* = 0.2130, *n* = 19). (B7) Following a 30‐min wash out of 10 µM 25CN‐NBOH, a significantly higher frequency of sEPSCs remained when compared to baseline (paired *t*‐test, *p* = 0.0092, *n* = 7). (C1) Representative recording from a mPFC neuron showing the presence of spontaneous inhibitory postsynaptic currents (sIPSCs) before and following exposure to bath applied 25CN‐NBOH at 10 µM in which conditions have been optimized to view inhibitory events as downward membrane deflections. sIPSCs were not altered in frequency or amplitude. (C2–3) There was also no significant effect on the frequency (Wilcoxon test, *p* = 0.7695, *n* = 10) or amplitude (Wilcoxon test, *p* = 0.7695, *n* = 10) of sIPSCs when evaluated across the population of cells. (D1) Representative traces showing the presence of sEPSCs from a mPFC neuron exposed to 30 nM MDL100907 and following exposure to bath applied 10 µM 25CN‐NBOH in MDL100907. (D2) In the presence of MDL100907, 10 µM 25CN‐NBOH failed to significantly increase the frequency of sEPSCs (paired *t*‐test, *p* = 0.8707, *n* = 9). (E1) Representative recordings of two different neurons in two different slices showing synaptic events in the mPFC in control conditions and following 1 h of incubation in 25CN‐NBOH. (E2–E3) Incubation in 10 µM 25CN‐NBOH did not result in significant differences in the frequency (Mann–Whitney test, *p* = 0.9310, *n* = 19) or amplitude (unpaired *t*‐test, *p* = 0.4195, *n* = 19) of sEPSCs. Data are represented as mean ± SEM. mPFC: medial prefrontal cortex; *n* = cells; sEPSCs: spontaneous excitatory postsynaptic currents; sEPSCs: inhibitory excitatory postsynaptic currents. *p < 0.05, **p < 0.01, and ***p < 0.00.

Our first studies evaluated the acute effect of 25CN‐NBOH on excitatory and inhibitory synaptic transmission directed to mPFC neurons (Figure 1A–C). Whole‐cell voltage‐clamp recordings were used to measure changes in the frequency and amplitude of spontaneous excitatory postsynaptic currents (sEPSCs) and spontaneous inhibitory postsynaptic currents (sIPSCs) after bath application of 25CN‐NBOH at concentrations of 200 nM and 10 µM. Our results demonstrated that 25CN‐NBOH at 200 nM failed to elicit a significant change in the frequency (Baseline: 1.79 ± 0.36 Hz, 200nM NBOH: 2.02 ± 0.49 Hz, *n* = 10/N_F_ = 2, N_M_ = 2; Figure 1B3) or the amplitude (Baseline: 10.72 ± 0.49 pA, 200nM NBOH: 10.72 ± 0.41 pA, *n* = 10/N_F_ = 2, N_M_ = 2; Figure 1B4) of sEPSCs. When evaluated by sex, neither females nor males showed a significant change in frequency (Females: Baseline: 1.63 ± 0.29 Hz, 200nM NBOH: 1.79 ± 0.47 Hz, *n* = 6/N_F_ = 2; *p* = 0.5484; Males: Baseline: 2.03 ± 0.85 Hz, 200nM NBOH: 2.36 ± 1.09 Hz, *n* = 4/ N_M_ = 2; *p* = 0.4119). The sEPSC amplitude was also not affected in either sex (Females: Baseline: 11.47 ± 0.53 pA, 200nM NBOH: 11.27 ± 0.46 pA, *n* = 6/N_F_ = 2; *p* = 0.5851; Males: Baseline: 9.58 ± 0.59 pA, 200nM NBOH: 9.90 ± 0.59 pA, *n* = 4/ N_M_ = 2; *p* = 0.0724).

In contrast, a higher concentration of 25CN‐NBOH (10 µM) resulted in a significant increase in the frequency of sEPSCs compared with baseline (Baseline: 2.24 ± 0.24 Hz, 10 µM NBOH: 2.75 ± 0.29 Hz, *n* = 19/N_F_ = 5, N_M_ = 4; Figure 1B5). The higher frequency was seen in females and males (Females: Baseline: 2.42 ± 0.36 Hz, 10 µM NBOH: 2.94 ± 0.38 Hz, *n* = 10/N_F_ = 5; *p* = 0.0279; Males: Baseline: 2.05 ± 0.31 Hz, 10µM NBOH: 2.53 ± 0.45 Hz, *n* = 9/N_M_ = 4; *p* = 0.0329). The amplitude of sEPSCs following exposure to 10 µM 25CN‐NBOH was not significantly different from baseline (Baseline: 11.09 ± 0.48 pA, 10 µM NBOH: 10.68 ± 0.52 pA, *n* = 19/N_F_ = 5, N_M_ = 4; Figure 1B6), which was reflected when males and females were analyzed separately (Females: Baseline: 10.46 ± 0.53 pA, 10 µM NBOH: 10.39 ± 0.80 pA, *n* = 10/ N_F_ = 5; *p* = 0.8827; Males: Baseline: 11.78 ± 0.80 pA, 10 µM NBOH: 11.00 ± 0.67 pA, *n* = 9/N_M_ = 4; *p* = 0.0699). Following a washout period of 30 min, the frequency of sEPSCs was still significantly higher than at baseline (Baseline: 2.14 ± 0.41Hz, 10 µM NBOH: 3.62 ± 0.75, *n* = 7/N_F_ = 2, N_M_ = 2; Figure 1B7).

Under conditions optimized for isolating sIPSCs, 10 µM 25CN‐NBOH did not significantly alter either the frequency of the inhibitory synaptic events (Baseline: 1.45 ± 0.26 Hz, 10 µM NBOH: 1.64 ± 0.36 Hz, *n* = 8/N_F_ = 2, N_M_ = 4; Figure 1C2) or the amplitude (Baseline: 14.43 ± 1.25 pA, 10 µM NBOH: 15.28 ± 1.66 pA, *n* = 8/N_F_ = 2, N_M_ = 4; Figure 1C3).

To determine whether the increased sEPSCs frequency involved 5‐HT_2A_ receptor activity, we applied the 5‐HT_2A_ receptor antagonist, 30 nM MDL100907 for 15 min prior to the application of 10 µM 25CN‐NBOH. We then evaluated whether the effects of 25CN‐NBOH on reducing the frequency of sEPSCs were affected. Our data show that the presence of MDL100907 abolished the effect of 10 µM 25CN‐NBOH on sEPSCs frequency (Baseline: 4.13 ± 0.79 Hz, 10 µM NBOH: 4.09 ± 0.75 Hz, *n* = 9/N_F_ = 2, N_M_ = 3; Figure 1D2), indicating that 5‐HT_2A_ receptor activation is necessary for 25CN‐NBOH's enhancement of excitatory transmission on mPFC pyramidal neurons.

Notably, the effect of 25CN‐NBOH on reducing the interval of sEPSCs was not present when the slice was exposed to 25CN‐NBOH for a longer period. When we incubated the slice in 25CN‐NBOH at 10 µM, in contrast to acute application, we did not note a significant difference in the frequency of sEPSCs (Baseline: 1.68 ± 0.21 Hz, *n* = 19/N_F_ = 4, N_M_ = 3; 10 µM NBOH: 1.93 ± 0.35 Hz, *n* = 19/N_F_ = 4, N_M_ = 4; Figure 1E2). We also did not note a significant change in amplitude (Baseline: 10.93 ± 0.39 pA, *n* = 19/N_F_ = 4, N_M_ = 3; 10 µM NBOH: 10.51 ± 0.34 pA, *n* = 19/N_F_ = 4, N_M_ = 4; Figure 1E3).

### 3.2 25CN‐NBOH Acute and Longer‐Term Exposure Reduces the Firing Rate of mPFC Neurons

**FIGURE 2:**
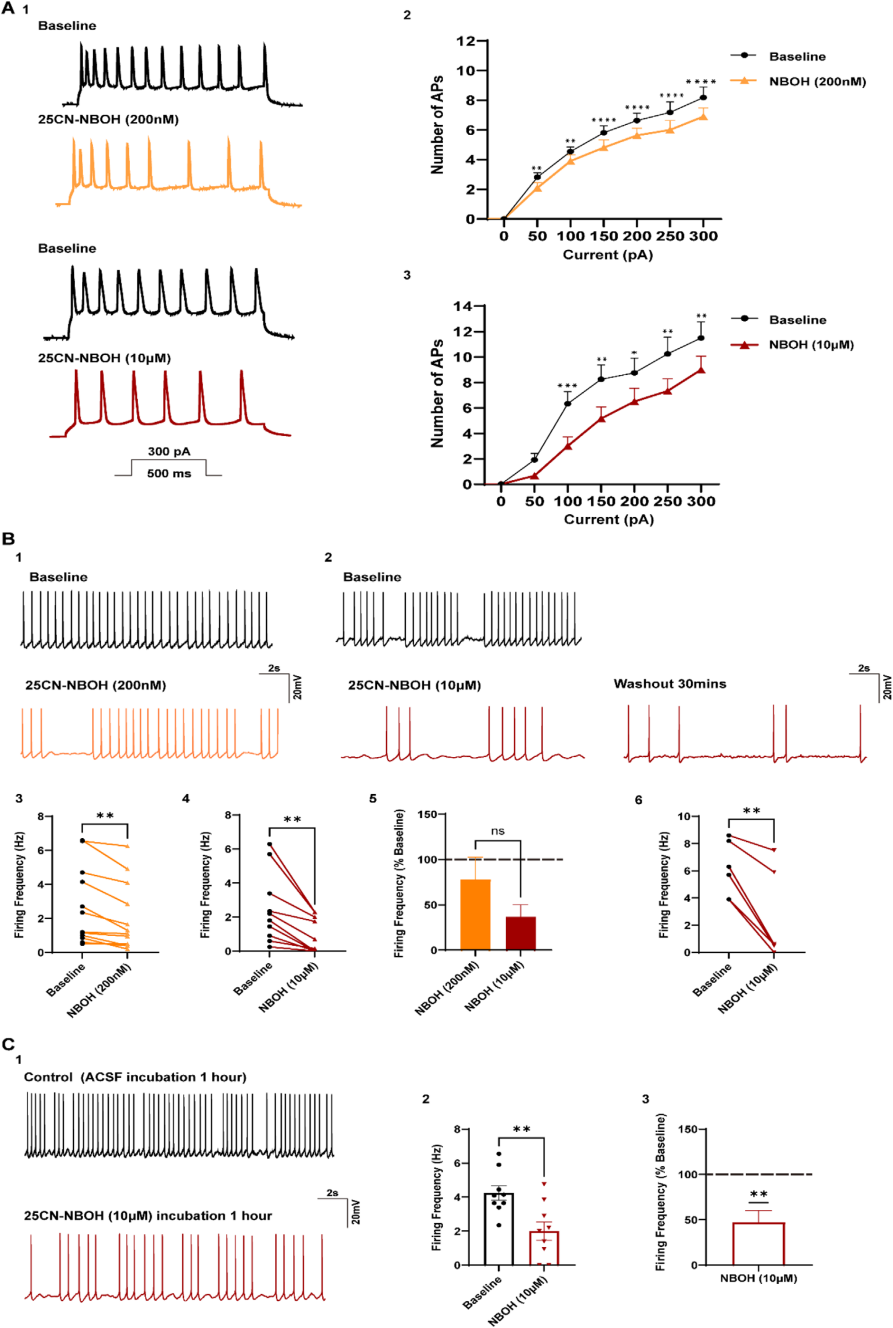
Decreased firing rates are induced by 25CN‐NBOH at both high and low concentrations, as well as with long‐term exposure. (A1) Representative recordings from a mPFC neuron showing elicitation of action potentials induced by injection of a 300‐pA current step at baseline and following exposure to 200 nM 25CN‐NBOH (yellow), and in another cell, following exposure to 10 µM 25CN‐NBOH (red). (A2–A3) In plotted input‐output curves from the population of cells, the number of action potentials as a function of the injected current reveal that 25CN‐NBOH significantly suppresses spiking both at the lower (2‐way ANOVA, *p* = 0.0004, *n* = 11) and higher concentrations (2‐way ANOVA, *p* = 0.0093, *n* = 12). (B1–B2) Representative recordings of spontaneous action potentials in individual mPFC neurons obtained by holding cells at the identical voltage sufficient to elicit firing during baseline conditions. These recordings reveal a decrease in firing rate during bath application of both 200 nM and 10 µM 25CN‐NBOH. The recording is shown following a 30‐min washout indicating persistence of the effect on firing. (B3) In the population of cells, the mean firing frequency of mPFC neurons was significantly reduced when comparing the firing frequency before and after 200 nM 25CN‐NBOH (Paired *t*‐test, *p* = 0.0019, *n* = 12). (B4) Spontaneous firing was reduced in the population of cells when 10 µM 25CN‐NBOH was applied (Wilcoxon test, *p* = 0.0020, *n* = 10). (B5) Normalization of the firing rate to control indicated both low and high concentrations suppressed the firing rate; however, there was not a significant effect of concentration on the magnitude of the effect (2‐way ANOVA, *p* = 0.5595, *n* = 10). (B6) In the population of cells, the firing rate was still significantly reduced following 30 min of washout of 10 µM 25CN‐NBOH (Paired *t*‐test, *p* = 0.0039, *n* = 6). (C1) Representative recording of spontaneous firing patterns from two different mPFC neurons at baseline and after 1‐h incubation with 10 µM 25CN‐NBOH showing decreased firing frequency. (C2) In the population of cells, the mean firing frequency in pyramidal neurons within the mPFC after 1‐h incubation with 10 µM 25CN‐NBOH was significantly lower than in baseline conditions (Wilcoxon test, *p* = 0.0156, *n* = 7). (C3) Normalization of the firing rate to control also showed a significant effect on the firing of incubation in 25CN‐NBOH (One sample *t*‐test, *p* = 0.0034, *n* = 9). Data are represented as mean ± SEM. mPFC: medial prefrontal cortex; *n* = cells; sEPSCs: excitatory postsynaptic currents. *p < 0.05, **p < 0.01, and ***p < 0.00.

To assess whether the change in excitatory synaptic activity is associated with a change in firing rate, we evaluated the effects of the two concentrations of 25CN‐NBOH in current‐clamp mode (Figure 2A1). Two protocols were used to elicit action potentials to evaluate the effects of 25CN‐NBOH on firing frequency. In the first protocol, firing was elicited by applying current steps from 0 to 300 pA in 50 pA increments. Under this protocol, both 200 nM (2‐way ANOVA, *p* = 0.0004, *n* = 11/N_F_ = 3, N_M_ = 2, Tukey's multiple comparison test, all current steps *p* ≤ 0.05, Figure 2A2) and 10 µM 25CN‐NBOH (2‐way ANOVA, *p* = 0.0093, *n* = 12/N_F_ = 3, N_M_ = 3, Tukey's multiple comparison test, all current steps *p* ≤ 0.05, except 50 pA, Figure 2A3), significantly suppressed the firing rate.

In the second protocol, the membrane potential was held at a potential sufficient for the initiation of spontaneous action potentials before and after application of 200 nM or 10 µM 25CN‐NBOH (Figure 2B1–B2). Under this protocol, 200 nM 25CN‐NBOH (Baseline: 2.70 ± 0.65 Hz; 200 nM NBOH: 2.05 ± 0.58 Hz, *n* = 12/N_F_ = 3, N_M_ = 3; Figure 2B3) and 10 µM 25CN‐NBOH (Baseline: 2.50 ± 0.65 Hz; 10 µM NBOH: 0.92 ± 0.33 Hz, *n* = 10/N_F_ = 2, N_M_ = 2; Figure 2B4) reduced the firing rate to 23.96 ± 2.67 % and 63.13 ± 13.04 %, respectively. A significant change in firing at both concentrations was seen in females (Baseline: 3.21 ± 0.97 Hz; 200 nM NBOH: 2.60 ± 0.83 Hz, *n* = 7 /N_F_ = 3, *p* = 0.0475; Baseline: 1.74 ± 0.51 Hz; 10 µM NBOH: 0.78 ± 0.45 Hz, *n* = 5/N_F_ = 2, *p* = 0.0092) and males (Baseline: 1.97 ± 0.78 Hz; 200 nM NBOH: 1.28 ± 0.73 Hz, *n* = 5/N_M_ = 3, *p* = 0.0241; Baseline: 3.25 ± 1.17 Hz; 10 µM NBOH: 1.06 ± 0.522 Hz, *n* = 5/N_M_ = 2, *p* = 0.0316). When the data were normalized to control, no significant difference was observed in the reduction in firing between the two concentrations (200 nM NBOH: 76.04 ± 21.59%; 10 µM NBOH: 36.87 ± 13.15%, Figure 2B5). Followed by 30 min of washout with ACSF, the effect of decreased firing rate elicited by 10 µM 25CN‐NBOH was still observed when compared with firing seen at baseline (Baseline: 6.10 ± 0.83 Hz,10 µM NBOH: 2.52 ± 1.34, *n* = 6/N_F_ = 2, N_M_ = 2; Figure 2B6). In addition, a 1‐h incubation with 10 µM 25CN‐NBOH significantly reduced the firing rate by 52.75 ± 2.75% under this protocol (Baseline: 4.24 ± 0.43 Hz; *n* = 9/N_F_ = 4, N_M_ = 2; 10 µM NBOH: 2.01 ± 0.54 Hz, *n* = 9/N_F_ = 3, N_M_ = 2; Figure 2C2–C3), suggesting persistence of this action.

Contrary to expectations based on previous literature and our synaptic data at higher concentrations, our findings showed that both low (200 nM) and high (10 µM) concentrations of 25CN‐NBOH significantly decreased the firing rate of mPFC neurons in both protocols (Figure 2A,B). Further, effects on firing persisted during a more chronic exposure, whereas effects of 10 µM 25CN‐NBOH on the frequency of synaptic events while persistent for 30 min post‐drug exposure were not seen with an hour exposure. Together with our sEPSCs data, these results suggest that 25CN‐NBOH exerts distinct effects on mPFC neurons: one effect likely presynaptic, and the other possibly directly on the postsynaptic cells.

### 3.3 The Suppression of Firing Rate Is Independent of the 5‐HT_2A_ Receptor and Involves Activation of M‐channel Current

**FIGURE 3:**
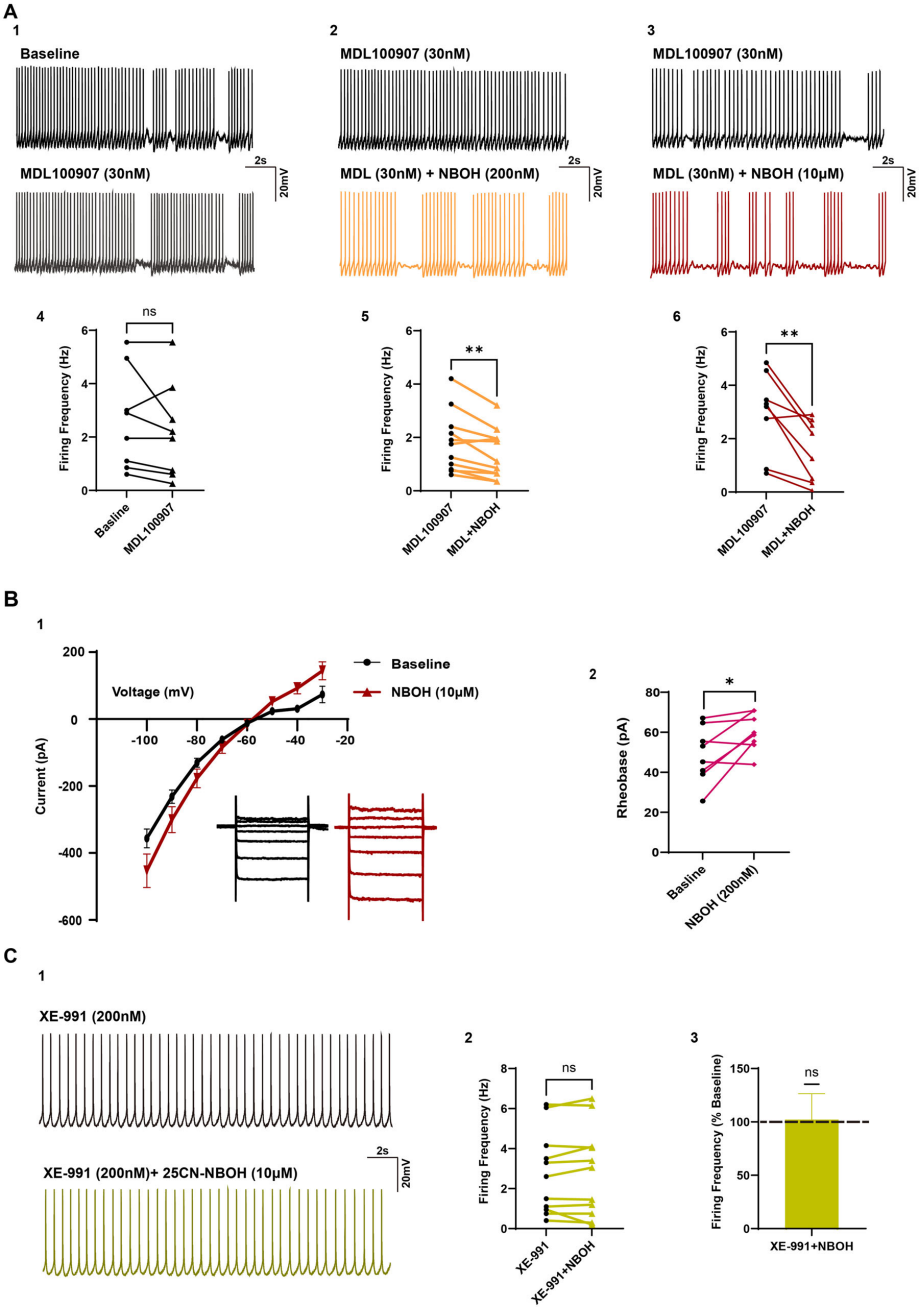
The suppression of action potentials is independent of the 5‐HT_2A_ receptor but is mediated by the M‐channel current. (A1) Representative recordings of action potentials from a pyramidal neuron in the mPFC held at the identical voltage sufficient to elicit firing under baseline conditions and following treatment with 30 nM MDL100907. (A2) Representative recordings of action potentials of a pyramidal neuron in 30 nM MDL100907 and after exposure to 200 nM 25CN‐NBOH in 30 nM MDL100907. (A3) Representative recordings of action potentials in another pyramidal neuron in 30 nM MDL100907 and after exposure to 10 µM 25CN‐NBOH in 30 nM MDL100907. (A4) While in some cells, a reduction in firing was noted when MDL100907 was applied alone, across the population of recorded mPFC neurons, there was not a significant change in firing rate (paired *t*‐test, *p* = 0.2604, *n* = 8). (A5) Paired data plots are shown from a population of cells reflecting a significant reduction in firing frequency following 200 nM 25CN‐NBOH in 30 nM MDL100907 (Wilcoxon *t*‐test, *p* = 0.0049, *n* = 11). (A6) Firing frequency plotted from a population of cells in the presence of MDL100907 alone and after co‐application with 10 µM 25CN‐NBOH reveals a significant reduction in firing frequency even in presence of the 5‐HT_2A_ receptor antagonist (Paired *t*‐test, *p* = 0.0082, *n* = 8). (B1) Current–voltage (I–V) relationship curves of mPFC neurons before (black) and after (red) 25CN‐NBOH application, showing crossing of the two curves near −60 mV, consistent with activation by 25CN‐NBOH of a potassium conductance. (B2) Rheobase protocols indicated that application of 200 nM 25CN‐NBOH induced a significant increase in the current required to elicit an action potential in pyramidal mPFC neurons (Paired *t*‐test, *p* = 0.0343, *n* = 8). (C1) Representative recordings of the action potentials in a mPFC neuron held at the identical voltage sufficient to elicit firing during bath application of XE‐991 (black) and after application of 10 µM 25CN‐NBOH in XE‐991 held at an identical voltage (yellow), indicating no significant change in firing frequency. (C2) In the population of mPFC cells, 10 µM 25CN‐NBOH failed to produce a significant change in firing frequency of mPFC neurons in the presence of XE‐991 (Paired *t*‐test, *p* = 0.6033 *n* = 11). (C3) Normalization of the firing rate to control also showed no significant effect in the presence of 25CN‐NBOH (One sample *t*‐test, *p* = 0.9320, *n* = 11). Data are represented as mean ± SEM. sEPSCs: spontaneous excitatory postsynaptic currents; mPFC: medial prefrontal cortex; *n* = cells. *p < 0.05, **p < 0.01, and ***p < 0.00.

To elucidate the mechanism underlying the effect of 25CN‐NBOH on decreased firing frequency, we investigated the involvement of 5‐HT_2A_ receptors using the 5‐HT_2A_ receptor antagonist MDL100907. Unexpectedly, the findings revealed that 30 nM MDL100907 failed to block the 25CN‐NBOH‐mediated suppression of the firing rate at the low (Baseline: 1.82 ± 0.34 Hz; 200 nM NBOH: 1.38 ± 0.28 Hz, *n* = 11/N_F_ = 2, N_M_ = 2; Figure 3A5) or high agonist concentrations (Baseline: 2.96 ± 0.54 Hz; 10 µM NBOH: 1.56 ± 0.41 Hz, *n* = 8/N_F_ = 1, N_M_ = 3; Figure 3A6). These data suggested that 25CN‐NBOH targets an effector that modifies neuronal excitability independently of the 5‐HT_2A_ receptor. To further explore the underlying mechanism, we first examined the current–voltage (I–V) relationship during 25CN‐NBOH application (Figure 3B1) and noted a reversal potential of the 25CN‐NBOH‐mediated current around −60 mV. When corrected for the liquid junction potential of 14 mV, the reversal potential suggested the involvement of a potassium conductance. We also evaluated rheobase and found that 25CN‐NBOH significantly increased this parameter (Baseline: 48.90 ± 4.93 pA; 200 nM NBOH: 59.95 ± 3.26 pA, *n* = 11/N_F_ = 2, N_M_ = 2; Figure 3B2). Together, our data suggested the possibility of involvement of M‐channels, which are known to regulate neuronal excitability by limiting repetitive firing and suppressing spontaneous firing (Brown and Passmore 2009; Marrion 1997). M‐channels play a profound role in pyramidal mPFC neuronal functionality where they have been shown to modify the firing kinetics through a decrease in spike frequency and a reduction in the inter‐spike interval between action potentials (Peng et al. 2017), which were consistent with our data. As further support of our hypothesis of a role of the M‐channel, a demonstration of a role for the M‐channel in 25CN‐NBOH effects on mPFC firing appeared in preprint (Ekins et al. 2023).

To evaluate whether the M‐channel activation contributes to the suppression of firing elicited by 25CN‐NBOH, we applied 25‐CN‐NBOH in the presence of the M‐channel blocker XE‐991. In the presence of 200 nM XE‐991, 25CN‐NBOH did not suppress the firing rate (Baseline: 2.773 ± 0.62 Hz; 10 µM NBOH: 2.83 ± 0.68 Hz, *n* = 11/N_F_ = 2, N_M_ = 3; Figure 3C2). Our data support the role of the M‐channel in the 25CN‐NBOH‐mediated action of suppressing the firing of action potentials.

When taken together, our data suggest that the effects of 25CN‐NBOH include actions at the 5HT_2A_ receptor resulting in enhancement of excitatory synaptic activity, as well as a postsynaptic action involving the M‐current, which is independent of 5‐HT_2A_ receptor activation.

## 4 Discussion

In this study, we evaluated the cellular effects of acute and long‐term exposure to 25CN‐NBOH, a selective 5‐HT_2A_ receptor agonist on mPFC pyramidal neurons in mouse brain slices. We found that 25CN‐NBOH enhances excitatory transmission through 5‐HT_2A_ receptor activation in a concentration‐dependent manner. Further, our data elucidated that neuronal firing was decreased via M‐channel activation that did not appear to involve the 5‐HT_2A_ receptor. In addition, when the slice was exposed to 25CN‐NBOH for 1‐h incubation, we discovered that while the effects on synaptic events were not present, the effect on reducing action potential firing was persistent. To date, a peer‐reviewed study of the cellular effect of 25CN‐NBOH has not appeared. During our studies, we became aware of a preprint by Schmitz et al. (2022), who reported in mouse brain slices that 200 nM 25CN‐NBOH enhanced firing in pyramidal mPFC neurons and did not affect sEPSCs. Our data are not in complete agreement with those findings. Here, we present our data, placing our work in the context not only of the Schmitz preprint but also in the context of a preprint by Ekins et al. (2023), which appeared later and reports data in concordance with our results.

Glutamatergic input arises to mPFC cells from multiple neural regions including the hippocampus, the amygdala, and the ventral tegmental area (Jefferson et al. 2021; Little and Carter 2012). All of these regions are involved in the control of mood and affective state, and the alteration of glutamatergic transmission from the mPFC to these regions has been implicated both in depression and in therapeutic approaches (Carlson et al. 2006; Vose and Stanton 2017). The 5‐HT_2A_ receptor is found on presynaptic terminals directed to mPFC cells (Becamel et al. 2017). The 25CN‐NBOH‐mediated increase in sEPSCs frequency is in agreement with previous studies showing that psychedelic substances such as LSD and DOI enhance glutamatergic transmission through 5‐HT_2A_ receptor activation, which may promote neuroplasticity and contribute to therapeutic effects (Aleksandrova and Phillips 2021; Muschamp et al. 2004; Ly et al. 2018; Scruggs et al. 2000; C. Zhang and Marek 2008). Further, our data also support recent findings suggesting that this action is concentration‐dependent. In a recent preprint, when tested at two different concentrations, the highest concentrations of 25CN‐NBOH, 10, and 100 µM, significantly increased the frequency of sEPSCs in mPFC neurons in mouse brain slices with no effect on amplitude; whereas, lower concentrations of 0.1 and 1 µM failed to have an effect on either frequency or amplitude of sEPSCs (Ekins et al. 2023). Similarly, in another preprint evaluating the effects of 25CN‐NBOH ex vivo in mouse mPFC neurons, 200 nM 25CN‐NBOH failed to induce any changes in sEPSCs frequency or amplitude (Schmitz et al. 2022). We found that the increase in sEPSCs frequency induced by 10 µM 25CN‐NBOH was blocked by the 5‐HT_2A_ receptor antagonist MDL100907. This aligns with unpublished findings (Ekins et al. 2023), where the 5‐HT_2A_ receptor antagonist ketanserin produced similar effects, and extends them as MDL100907 has been suggested as a more selective 5‐HT_2A_ receptor antagonist than ketanserin which exhibits greater off‐target effects (Casey et al. 2022). When taken together, our study and the work of others suggest that 25CN‐NBOH can induce increases in the release of glutamate directed to mPFC pyramidal cells, putatively through a presynaptic action involving the 5‐HT_2A_ receptor. However, the effect is concentration‐dependent suggesting that a certain level of 5‐HT_2A_ receptor occupancy is required to stimulate glutamatergic presynaptic inputs directed to mPFC cells.

While mPFC neurons receive GABAergic input (Hubner et al. 2014; Radley et al. 2009), and 5‐HT_2A_ receptors are present in these cells (Nocjar et al. 2015; Santana et al. 2004), we did not see a significant effect of the higher concentration of 25CN‐NBOH on inhibitory synaptic activity directed to mPFC neurons. This is in agreement with another study in preprint, which used a lower concentration of 25CN‐NBOH (Schmitz et al. 2022). When taken together, the data suggest that at the concentrations tested, 5‐HT_2A_ receptors are not activated on inhibitory presynaptic input by this selective receptor agonist. However, it is possible that at higher concentrations, these neurons could be activated, leading to an increase in inhibitory drive to mPFC cells. Maintaining the proper balance between excitation and inhibition is critical for the normal function of cortical circuits, and it would be interesting in future studies to explore if concentrations of 25CN‐NBOH exceed those evaluated, or if other psychedelics targeting the 5‐HT_2A_ receptor exert actions on inhibitory synaptic drive (Nelson and Turrigiano 2008; Turrigiano 2011).

Effects on sEPSCs were still present during 30 min of washout, suggesting some long‐term alteration in synaptic activity following drug exposure. However, incubation data suggested that effects on synaptic activity did not persist during constant exposure. Our data are novel, as other studies have not reported this effect. This is interesting in light of the glutamatergic plasticity believed to be induced by psychedelics. This plasticity can be pre or postsynaptically mediated. Our data would suggest that at the concentration used and under the exposure protocols evaluated, some putative presynaptic plasticity is present following acute exposure. However, if exposure is constant, this plasticity is not evident, perhaps due to receptor desensitization or internalization (Gainetdinov et al. 2004). Future studies should determine the duration of time after acute exposure that changes in the frequency of sEPSCs persist, and whether changes seen are pre‐ or post‐synaptically mediated.

We found that during the application, and washout of 25CN‐NBOH, reductions in the firing frequency of mPFC neurons were elicited through a persistent action on M‐channels, which did not involve activation of the 5‐HT_2A_ receptor. The inhibition persisted following the incubation of 25CN‐NBOH. The inhibition of firing by a selective 5‐HT_2A_ receptor agonist challenges the prevailing hypothesis that psychedelics uniformly enhance the neuronal excitability of pyramidal cells, leading to an increase in glutamate release to target regions (Aleksandrova and Phillips 2021; Marek 2018; Muschamp et al. 2004). However, earlier studies have shown that psychedelics including LSD can suppress the firing of serotonin‐containing neurons within the dorsal raphe nucleus in rats (Mccall 1982) and other studies showed that LSD attenuated the firing activity both in the cortex and hippocampus, which was proposed to underlie beneficial actions on obsessive‐compulsive disorder (Zghoul and Blier 2003). 5‐HT_2A_ receptor activation has been shown to influence sodium channels within the mPFC, which could lead to decreases in excitability (Carr et al. 2002, 2003, 2002, 2003). Further, LSD has been shown to hyperpolarize serotonergic neurons of the dorsal raphe by increasing potassium conductances (Aghajanian and Lakoski 1984). Our data are in alignment with the latter and suggest that while 25CN‐NBOH is a highly selective 5‐HT_2A_ receptor agonist, it also activates other effectors similar to those activated by psychedelics. This action may play a role in potential therapeutic actions seen with 25CN‐NBOH in preclinical studies (Odland et al. 2021).

Our results contrast with other data in a preprint in which an enhancement of 25CN‐NBOH on the firing rate of mPFC neurons was presented (Schmitz et al. 2022). We cannot account for the discrepancy between our results and those of this study. We do not think the differences in strain of mice used in the two different studies explain the discrepant results. While Schmitz used C57 mice and we used NMRI animals, in another preprint, effects on the firing of 25CN‐NBOH were evaluated in C57 mice, and results similar to ours were obtained (Ekins et al. 2023). Although we did not observe a concentration‐dependent effect of 25CN‐NBOH on firing rates with the two concentrations we evaluated, in the latter study, while suppression of firing rate was elicited at all concentrations, the magnitude of the effect was concentration‐dependent when using 25CN‐NBOH (Ekins et al. 2023). Taken together with other studies, our data suggest that the effects of psychedelics on neuronal excitability are complex and may depend on multiple factors, including concentration and specific receptor interactions.

Our study has limitations. While we found no differences based on sex in the change in frequency of sEPSCs and action potential firing affected by 25CN‐NBOH, a focused evaluation of whether sex differences exist in other cellular effects of 25CN‐NBOH should be conducted as sex‐based differences in molecular players, which could be involved in membrane and synaptic effects of 25CN‐NBOH have been noted (Giacometti and Barker 2020; Jaster et al. 2022; Sakamoto and Kurokawa 2019; Shadani et al. 2024). Further, we evaluated effects within a limited age range, and as relevant molecular players also change across development, evaluation of cellular effects in mPFC neurons from older animals could vary from those seen in younger animals, and this should be expressly examined. Finally, as ours is an ex vivo study, it is difficult to compare the concentrations we used with those that would be experienced in vivo. Based on published pharmacokinetic data, 25CN‐NBOH is stable (Jensen et al. 2017). Concentrations of 200 nM are obtained in the brains of mice following s.c. administration of 3 mg/kg and behavioral actions in mice and rats are seen following s.c. and i.p. administration of up to 1–2 mg/kg, which would correspond to a concentration of approximately 100 nM, which matches well with the EC50 at the 5HT_2A_ receptor of 1 nM (Jensen et al. 2017). Accordingly, while data collected with 200 nM exposures could be realistic, the results we obtained when using 10 µM, might not be representative of exposures achieved in rodents dosed with 1‐3 mg/kg, or at that concentration, it should be considered that effects could extend beyond the 5HT_2A_ receptor. Regardless, our data highlight the necessity to consider differential effects based on concentration.

While some discrepancies between our data and those in preprint exist, we believe for several reasons that it is important to make our data available: (1) the current dogma would suggest an increase in firing of mPFC neurons is elicited by 5HT_2A_ receptor agonists, and as a specific 5HT_2A_ receptor agonist, this would be assumed as a mechanism of action of 25CN‐NBOH as well. Clarifying that in fact, in two independent studies, 25CN‐NBOH results in a reduction in firing at several different concentrations is important to consider when incorporating actions of this drug into therapeutic models. (2) Replication of studies is critical if we are to advance drug development with as little time as possible spent pursuing results, which do not represent the correct biology. Specific to 25CN‐NBOH, it appears there is a conflict in the preprint literature, which our data can perhaps assist in reconciling. Further, our data extend the other studies in the preprint literature by showing that effects on action potential firing frequency persist in incubations with 25CN‐NBOH, which was in contrast to effects on synaptic activity.

In summary, our findings reveal that 25CN‐NBOH has a dual action on mPFC neurons, which highlights the need to consider both excitatory and inhibitory processes when evaluating the effects of psychedelics. Further research is necessary to determine whether balancing these opposing actions contributes to the therapeutic or hallucinogenic effects of psychedelic compounds. Ultimately, understanding the cellular effects of these drugs could facilitate the development of more specific and targeted psychedelic compounds with applications beyond mental health, potentially extending to other cognitive‐based disorders.

## Author Contributions

All authors conceptualized, investigated, data curated, formally analyzed, and visualized the study. Y.W. acquired and curated data. K.A.K. and Y.W. acquired funding for the study. All authors drafted, revised, reviewed and edited the article critically and all authors approved the final revision. The funding source was not involved in the study design, the collection, analysis or interpretation of the data

## Conflicts of Interest

The authors declare no conflicts of interest.

5

## Data Availability

Data is held in a repository at the University of Copenhagen and is available upon request made to the corresponding author

## References

[syn70014-bib-0001] Aghajanian, G. K. , and J. M. Lakoski . 1984. “Hyperpolarization of Serotonergic Neurons by Serotonin and LSD: Studies in Brain Slices Showing Increased K+ Conductance.” Brain Research 305, no. 1: 181–185. 10.1016/0006-8993(84)91137-5. 6331598

[syn70014-bib-0002] Aleksandrova, L. R. , and A. G. Phillips . 2021. “Neuroplasticity as a Convergent Mechanism of Ketamine and Classical Psychedelics.” Trends in Pharmacological Sciences 42, no. 11: 929–942. 10.1016/j.tips.2021.08.003. 34565579

[syn70014-bib-0003] Artigas, F. 2013. “Serotonin Receptors Involved in Antidepressant Effects.” Pharmacology & Therapeutics 137, no. 1: 119–131. 10.1016/j.pharmthera.2012.09.006. 23022360

[syn70014-bib-0004] Becamel, C. , C. Berthoux , A. Barre , and P. Marin . 2017. “Growing Evidence for Heterogeneous Synaptic Localization of 5‐HT2A Receptors.” ACS Chemical Neuroscience 8, no. 5: 897–899. 10.1021/acschemneuro.6b00409. 28459524

[syn70014-bib-0005] Brown, D. A. , and G. M. Passmore . 2009. “Neural KCNQ (Kv7) Channels.” British Journal of Pharmacology 156, no. 8: 1185–1195. 10.1111/j.1476-5381.2009.00111.x. 19298256 PMC2697739

[syn70014-bib-0006] Canal, C. E. 2018. “Serotonergic Psychedelics: Experimental Approaches for Assessing Mechanisms of Action.” In New Psychoactive Substances: Pharmacology, Clinical, Forensic and Analytical Toxicology, edited by H. H. Maurer and S. D. Brandt , 227–260. Springer. 10.1007/164_2018_107. PMC6136989 29532180

[syn70014-bib-0007] Carlson, P. J. , J. B. Singh , C. A. Zarate, Jr. , W. C. Drevets , and H. K. Manji . 2006. “Neural Circuitry and Neuroplasticity in Mood Disorders: Insights for Novel Therapeutic Targets.” NeuroRX 3, no. 1: 22–41. 10.1016/j.nurx.2005.12.009. 16490411 PMC3593361

[syn70014-bib-0008] Carr, D. B. , D. C. Cooper , S. L. Ulrich , N. Spruston , and D. J. Surmeier . 2002. “Serotonin Receptor Activation Inhibits Sodium Current and Dendritic Excitability in Prefrontal Cortex via a Protein Kinase C‐Dependent Mechanism.” Journal of Neuroscience 22, no. 16: 6846–6855. 10.1523/JNEUROSCI.22-16-06846.2002. 12177182 PMC6757866

[syn70014-bib-0009] Carr, D. B. , M. Day , A. R. Cantrell , et al. 2003. “Transmitter Modulation of Slow, Activity‐Dependent Alterations in Sodium Channel Availability Endows Neurons With a Novel Form of Cellular Plasticity.” Neuron 39, no. 5: 793–806. 10.1016/s0896-6273(03)00531-2. 12948446

[syn70014-bib-0010] Casey, A. B. , M. Cui , R. G. Booth , and C. E. Canal . 2022. ““Selective” Serotonin 5‐HT Receptor Antagonists.” Biochemical Pharmacology 200: 115028. 10.1016/j.bcp.2022.115028. 35381208 PMC9252399

[syn70014-bib-0011] de Vos, C. M. H. , N. L. Mason , and K. P. C. Kuypers . 2021. “Psychedelics and Neuroplasticity: A Systematic Review Unraveling the Biological Underpinnings of Psychedelics.” Frontiers in Psychiatry 12: 724606. 10.3389/fpsyt.2021.724606. 34566723 PMC8461007

[syn70014-bib-0012] Drevets, W. C. , J. L. Price , and M. L. Furey . 2008. “Brain Structural and Functional Abnormalities in Mood Disorders: Implications for Neurocircuitry Models of Depression.” Brain Structure and Function 213, no. 1–2: 93–118. 10.1007/s00429-008-0189-x. 18704495 PMC2522333

[syn70014-bib-0013] Ekins, T. G. , I. Brooks , S. Kailasa , et al. 2023. “Cellular Rules Underlying Psychedelic Control of Prefrontal Pyramidal Neurons.” Preprint, bioRxiv, October 23. 10.1101/2023.10.20.563334.

[syn70014-bib-0014] Fantegrossi, W. E. , B. W. Gray , J. M. Bailey , D. A. Smith , M. Hansen , and J. L. Kristensen . 2015. “Hallucinogen‐Like Effects of 2‐([2‐(4‐cyano‐2,5‐dimethoxyphenyl) ethylamino]Methyl)Phenol (25CN‐NBOH), a Novel N‐benzylphenethylamine With 100‐fold Selectivity for 5‐HT(2)A Receptors, in Mice.” Psychopharmacology 232, no. 6: 1039–1047. 10.1007/s00213-014-3739-3. 25224567 PMC4339409

[syn70014-bib-0015] Gainetdinov, R. R. , R. T. Premont , L. M. Bohn , R. J. Lefkowitz , and M. G. Caron . 2004. “Desensitization of G Protein‐Coupled Receptors and Neuronal Functions.” Annual Review of Neuroscience 27: 107–144. 10.1146/annurev.neuro.27.070203.144206. 15217328

[syn70014-bib-0016] Giacometti, L. L. , and J. M. Barker . 2020. “Sex Differences in the Glutamate System: Implications for Addiction.” Neuroscience and Biobehavioral Reviews 113: 157–168. 10.1016/j.neubiorev.2020.03.010. 32173404 PMC7225077

[syn70014-bib-0017] Gouveia, K. , and J. L. Hurst . 2013. “Reducing Mouse Anxiety During Handling: Effect of Experience With Handling Tunnels.” PLoS ONE 8, no. 6: e66401. 10.1371/journal.pone.0066401. 23840458 PMC3688777

[syn70014-bib-0018] Hansen, M. , K. Phonekeo , J. S. Paine , et al. 2014. “Synthesis and Structure‐Activity Relationships of‐Benzyl Phenethylamines as 5‐HT Agonists.” ACS Chemical Neuroscience 5, no. 3: 243–249. 10.1021/cn400216u. 24397362 PMC3963123

[syn70014-bib-0019] Hubner, C. , D. Bosch , A. Gall , A. Luthi , and I. Ehrlich . 2014. “Ex Vivo Dissection of Optogenetically Activated mPFC and Hippocampal Inputs to Neurons in the Basolateral Amygdala: Implications for Fear and Emotional Memory.” Frontiers in Behavioral Neuroscience 8: 64. 10.3389/fnbeh.2014.00064. 24634648 PMC3943336

[syn70014-bib-0020] Jaster, A. M. , J. Younkin , T. Cuddy , et al. 2022. “Differences Across Sexes on Head‐Twitch Behavior and 5‐HT Receptor Signaling in C57BL/6J Mice.” Neuroscience Letters 788: 136836. 10.1016/j.neulet.2022.136836. 35963476 PMC10114867

[syn70014-bib-0021] Jefferson, T. , C. J. Kelly , and M. Martina . 2021. “Differential Rearrangement of Excitatory Inputs to the Medial Prefrontal Cortex in Chronic Pain Models.” Frontiers in Neural Circuits 15: 791043. 10.3389/fncir.2021.791043. 35002635 PMC8738091

[syn70014-bib-0022] Jensen, A. A. , J. D. McCorvy , S. Leth‐Petersen , et al. 2017. “Detailed Characterization of the in Vitro Pharmacological and Pharmacokinetic Properties of ‐(2‐Hydroxybenzyl)‐2,5‐Dimethoxy‐4‐Cyanophenylethylamine (25CN‐NBOH), a Highly Selective and Brain‐Penetrant 5‐HT Receptor Agonist.” Journal of Pharmacology and Experimental Therapeutics 361, no. 3: 441–453. 10.1124/jpet.117.239905. 28360333

[syn70014-bib-0023] Koenigs, M. , and J. Grafman . 2009. “The Functional Neuroanatomy of Depression: Distinct Roles for Ventromedial and Dorsolateral Prefrontal Cortex.” Behavioural Brain Research 201, no. 2: 239–243. 10.1016/j.bbr.2009.03.004. 19428640 PMC2680780

[syn70014-bib-0024] Little, J. P. , and A. G. Carter . 2012. “Subcellular Synaptic Connectivity of Layer 2 Pyramidal Neurons in the Medial Prefrontal Cortex.” Journal of Neuroscience 32, no. 37: 12808–12819. 10.1523/JNEUROSCI.1616-12.2012. 22973004 PMC3490687

[syn70014-bib-0025] Ly, C. , A. C. Greb , L. P. Cameron , et al. 2018. “Psychedelics Promote Structural and Functional Neural Plasticity.” Cell Reports 23, no. 11: 3170–3182. 10.1016/j.celrep.2018.05.022. 29898390 PMC6082376

[syn70014-bib-0026] Marek, G. J. 2018. “Interactions of Hallucinogens With the Glutamatergic System: Permissive Network Effects Mediated through Cortical Layer V Pyramidal Neurons.” Behavioral Neurobiology of Psychedelic Drugs 36: 107–135. 10.1007/7854_2017_480. 28831734

[syn70014-bib-0027] Marrion, N. V. 1997. “Control of M‐Current.” Annual Review of Physiology 59: 483–504. 10.1146/annurev.physiol.59.1.483. 9074774

[syn70014-bib-0028] Martin, D. A. , and C. D. Nichols . 2018. “The Effects of Hallucinogens on Gene Expression.” In Behavioral Neurobiology of Psychedelic Drugs, edited by A. L. Halberstadt , F. X. Vollenweider , and D. E. Nichols , 137–158. Springer. 10.1007/7854_2017_479. 28677095

[syn70014-bib-0029] Martín‐Ruiz, R. , V. Puig , P. Celada , et al. 2002. “Control of Serotonergic Function in Medial Prefrontal Cortex by Serotonin‐2A Receptors Through a Glutamate‐Dependent Mechanism.” Journal of Neuroscience 21, no. 24: 9856–9866. 10.1523/JNEUROSCI.21-24-09856.2001 PMC6763049 11739593

[syn70014-bib-0030] Mccall, R. B. 1982. “Neurophysiological Effects of Hallucinogens on Serotonergic Neuronal Systems.” Neuroscience and Biobehavioral Reviews 6, no. 4: 509–514. 10.1016/0149-7634(82)90033-1. 7177511

[syn70014-bib-0031] Moliner, R. , M. Girych , C. A. Brunello , et al. 2023. “Psychedelics Promote Plasticity by Directly Binding to BDNF Receptor TrkB.” Nature Neuroscience 26, no. 6: 1032–1041. 10.1038/s41593-023-01316-5. 37280397 PMC10244169

[syn70014-bib-0032] Muschamp, J. W. , M. J. Regina , E. M. Hull , J. C. Winter , and R. A. Rabin . 2004. “Lysergic Acid Diethylamide and [‐]‐2,5‐Dimethoxy‐4‐Methylamphetamine Increase Extracellular Glutamate in Rat Prefrontal Cortex.” Brain Research 1023, no. 1: 134–140. 10.1016/j.brainres.2004.07.044. 15364028

[syn70014-bib-0033] Nelson, S. B. , and G. G. Turrigiano . 2008. “Strength Through Diversity.” Neuron 60, no. 3: 477–482. 10.1016/j.neuron.2008.10.020. 18995822 PMC4919814

[syn70014-bib-0034] Nocjar, C. , K. D. Alex , A. Sonneborn , A. I. Abbas , B. L. Roth , and E. A. Pehek . 2015. “Serotonin‐2C and ‐2a Receptor Co‐Expression on Cells in the Rat Medial Prefrontal Cortex.” Neuroscience 297: 22–37. 10.1016/j.neuroscience.2015.03.050. 25818050 PMC4595040

[syn70014-bib-0035] Nutt, D. , D. Erritzoe , and R. Carhart‐Harris . 2020. “Psychedelic Psychiatry's Brave New World.” Cell 181, no. 1: 24–28. 10.1016/j.cell.2020.03.020. 32243793

[syn70014-bib-0036] Odland, A. U. , L. Jessen , J. L. Kristensen , C. M. Fitzpatrick , and J. T. Andreasen . 2021. “The 5‐Hydroxytryptamine 2A Receptor Agonists DOI and 25CN‐NBOH Decrease Marble Burying and Reverse 8‐OH‐DPAT‐Induced Deficit in Spontaneous Alternation.” Neuropharmacology 183: 107838. 10.1016/j.neuropharm.2019.107838. 31693871

[syn70014-bib-0037] Peng, H. , X. L. Bian , F. C. Ma , and K. W. Wang . 2017. “Pharmacological Modulation of the Voltage‐Gated Neuronal Kv7/KCNQ/M‐Channel Alters the Intrinsic Excitability and Synaptic Responses of Pyramidal Neurons in Rat Prefrontal Cortex Slices.” Acta Pharmacologica Sinica 38, no. 9: 1248–1256. 10.1038/aps.2017.72. 28603289 PMC5589969

[syn70014-bib-0038] Puig, M. V. , and A. T. Gulledge . 2011. “Serotonin and Prefrontal Cortex Function: Neurons, Networks, and Circuits.” Molecular Neurobiology 44, no. 3: 449–464. 10.1007/s12035-011-8214-0. 22076606 PMC3282112

[syn70014-bib-0039] Radley, J. J. , K. L. Gosselink , and P. E. Sawchenko . 2009. “A Discrete GABAergic Relay Mediates Medial Prefrontal Cortical Inhibition of the Neuroendocrine Stress Response.” Journal of Neuroscience 29, no. 22: 7330–7340. 10.1523/JNEUROSCI.5924-08.2009. 19494154 PMC2743123

[syn70014-bib-0040] Rorsted, E. M. , A. A. Jensen , G. Smits , K. Frydenvang , and J. L. Kristensen . 2024. “Discovery and Structure‐Activity Relationships of 2,5‐Dimethoxyphenylpiperidines as Selective Serotonin 5‐HT Receptor Agonists.” Journal of Medicinal Chemistry 67, no. 9: 7224–7244. 10.1021/acs.jmedchem.4c00082. 38648420 PMC11089506

[syn70014-bib-0041] Sakamoto, K. , and J. Kurokawa . 2019. “Involvement of sex hormonal regulation of K(+) channels in electrophysiological and contractile functions of muscle tissues.” J Pharmacol Sci 139, no. 4: 259–265. 10.1016/j.jphs.2019.02.009. 30962088

[syn70014-bib-0042] Santana, N. , A. Bortolozzi , J. Serrats , G. Mengod , and F. Artigas . 2004. “Expression of Serotonin1A and Serotonin2A Receptors in Pyramidal and GABAergic Neurons of the Rat Prefrontal Cortex.” Cerebral Cortex 14, no. 10: 1100–1109. 10.1093/cercor/bhh070. 15115744

[syn70014-bib-0043] Schmitz, G. P. , Y.‐T. Chiu , G. M. König , et al. 2022. “Psychedelic Compounds Directly Excite 5‐HT2A Layer 5 Pyramidal Neurons in the Prefrontal Cortex Through a 5‐HT2A Gq ‐Mediated Activation Mechanism.” Preprint, bioRxiv, November 15. 10.1101/2022.11.15.516655.

[syn70014-bib-0044] Scruggs, J. L. , S. Patel , M. Bubser , and A. Y. Deutch . 2000. “DOI‐Induced Activation of the Cortex: Dependence on 5‐HT2A Heteroceptors on Thalamocortical Glutamatergic Neurons.” Journal of Neuroscience 20, no. 23: 8846–8852. 10.1523/JNEUROSCI.20-23-08846.2000. 11102493 PMC6773058

[syn70014-bib-0045] Shadani, S. , K. Conn , Z. B. Andrews , and C. J. Foldi . 2024. “Potential Differences in Psychedelic Actions Based on Biological Sex.” Endocrinology 165, no. 8: bqae083. 10.1210/endocr/bqae083. 38980913 PMC11259856

[syn70014-bib-0046] Tullis, P. 2021. “The Rise of Psychedelic Psychiatry.” Nature 589, no. 7843: 506–509. 10.1038/d41586-021-00187-9. 33505033

[syn70014-bib-0047] Turrigiano, G. 2011. “Too Many Cooks? Intrinsic and Synaptic Homeostatic Mechanisms in Cortical Circuit Refinement.” Annual Review of Neuroscience 34: 89–103. 10.1146/annurev-neuro-060909-153238. 21438687

[syn70014-bib-0048] van Aerde, K. I. , and D. Feldmeyer . 2015. “Morphological and Physiological Characterization of Pyramidal Neuron Subtypes in Rat Medial Prefrontal Cortex.” Cerebral Cortex 25, no. 3: 788–805. 10.1093/cercor/bht278. 24108807

[syn70014-bib-0049] van Elk, M. , and D. B. Yaden . 2022. “Pharmacological, Neural, and Psychological Mechanisms Underlying Psychedelics: A Critical Review.” Neuroscience and Biobehavioral Reviews 140: 104793. 10.1016/j.neubiorev.2022.104793. 35878791

[syn70014-bib-0050] Vargas, M. V. , L. E. Dunlap , C. Y. Dong , et al. 2023. “Psychedelics Promote Neuroplasticity Through the Activation of Intracellular 5‐HT2A Receptors.” Science 379, no. 6633: 700–706. 10.1126/science.adf0435. 36795823 PMC10108900

[syn70014-bib-0051] Vose, L. R. , and P. K. Stanton . 2017. “Synaptic Plasticity, Metaplasticity and Depression.” Current Neuropharmacology 15, no. 1: 71–86. 10.2174/1570159x14666160202121111. 26830964 PMC5327460

[syn70014-bib-0052] Zghoul, T. , and P. Blier . 2003. “Enhancing Action of LSD on Neuronal Responsiveness to Serotonin in a Brain Structure Involved in Obsessive‐compulsive Disorder.” International Journal of Neuropsychopharmacology 6, no. 1: 13–21. 10.1017/S1461145702003218. 12899732

[syn70014-bib-0053] Zhang, C. , and G. J. Marek . 2008. “AMPA Receptor Involvement in 5‐Hydroxytryptamine2A Receptor‐Mediated Pre‐Frontal Cortical Excitatory Synaptic Currents and DOI‐Induced Head Shakes.” Progress in Neuro‐Psychopharmacology & Biological Psychiatry 32, no. 1: 62–71. 10.1016/j.pnpbp.2007.07.009. 17728034

[syn70014-bib-0054] Zhang, G. , and R. W. Stackman Jr. . 2015. “The Role of Serotonin 5‐HT2A Receptors in Memory and Cognition.” Frontiers in Pharmacology 6: 225. 10.3389/fphar.2015.00225. 26500553 PMC4594018

[syn70014-bib-0055] Zieba, A. , P. Stepnicki , D. Matosiuk , and A. A. Kaczor . 2022. “Overcoming Depression With 5‐HT Receptor Ligands.” International Journal of Molecular Sciences 23, no. 1: 10. 10.3390/ijms23010010. PMC8744644 35008436

